# Influenza A, Influenza B, human respiratory syncytial virus and SARSCoV-2 molecular diagnostics and epidemiology in the post COVID-19 era

**DOI:** 10.1186/s12931-024-02862-7

**Published:** 2024-06-05

**Authors:** Manca Luštrek, Zala Cesar, Alen Suljič, Rok Kogoj, Nataša Knap, Monika Jevšnik Virant, Tina Uršič, Miroslav Petrovec, Tatjana Avšič-Županc, Miša Korva

**Affiliations:** Institute of Microbiology and Immunology, Faculty of Medicine, University of Ljubljana, Zaloška cesta 4, Ljubljana, 1000 Slovenia

**Keywords:** SARS-CoV-2, Flu-A/B, HRSV, rtRT-PCR, Multiplex, Co-detections, Turnaround-time

## Abstract

**Background:**

The concurrent circulation of SARS-CoV-2 with other respiratory viruses is unstoppable and represents a new diagnostic reality for clinicians and clinical microbiology laboratories. Multiplexed molecular testing on automated platforms that focus on the simultaneous detection of multiple respiratory viruses in a single tube is a useful approach for current and future diagnosis of respiratory infections in the clinical setting.

**Methods:**

Two time periods were included in the study: from February to April 2022, an early 2022 period, during the gradual lifting of COVID-19 prevention measures in the country, and from October 2022 to April 2023, the 2022/23 respiratory infections season. We analysed a total of 1,918 samples in the first period and 18,131 respiratory samples in the second period using a multiplex molecular assay for the simultaneous detection of Influenza A (Flu-A), Influenza B (Flu-B), Human Respiratory Syncytial Virus (HRSV) and SARS-CoV-2.

**Results:**

The results from early 2022 showed a strong dominance of SARS-CoV-2 infections with 1,267/1,918 (66.1%) cases. Flu-A was detected in 30/1,918 (1.6%) samples, HRSV in 14/1,918 (0.7%) samples, and Flu-B in 2/1,918 (0.1%) samples. Flu-A/SARS-CoV-2 co-detections were observed in 11/1,267 (0.9%) samples, and HRSV/SARS-CoV-2 co-detection in 5/1,267 (0.4%) samples. During the 2022/23 winter respiratory season, SARS-CoV-2 was detected in 1,738/18,131 (9.6%), Flu-A in 628/18,131 (3.5%), Flu-B in 106/18,131 (0.6%), and HRSV in 505/18,131 (2.8%) samples. Interestingly, co-detections were present to a similar extent as in early 2022.

**Conclusion:**

The results show that the multiplex molecular approach is a valuable tool for the simultaneous laboratory diagnosis of SARS-CoV-2, Flu-A/B, and HRSV in hospitalized and outpatients. Infections with Flu-A/B, and HRSV occurred shortly after the COVID-19 control measures were lifted, so a strong reoccurrence of various respiratory infections and co-detections in the post COVID-19 period was to be expected.

## Introduction

The outbreak of SARS-CoV-2, which led to a global pandemic, required the immediate development of new laboratory tests [1]. Population-level testing relied heavily on molecular testing, which has been conducted on a large scale and with high throughput worldwide in recent years. During the COVID-19 pandemic, the main focus of diagnostic laboratories has been on testing for SARS-CoV-2 infections, on symptomatic patients who needed clinical treatment and on the general population to identify infectious individuals who needed to be isolated to prevent the spread of the virus [2]. To combat the pandemic, governments deployed COVID-19 control measures that not only affected the transmission of SARSCoV-2, but also limited the circulation of other respiratory pathogens such as influenza A (Flu-A), influenza B (Flu-B) and human respiratory syncytial virus (HRSV). Large outbreaks of influenza and HRSV could reoccur in the coming years, as a significant proportion of the population has not been exposed to these pathogens for more than 2 years. Therefore, healthcare systems should be aware of a possible higher burden of respiratory infections in the future and plan their testing strategy accordingly, especially because it is obvious that SARS-CoV-2 infections overlap with other respiratory infections [3] and are likely to persist.

The co-occurrence of SARS-CoV-2 with other respiratory viral infections and possible codetection poses a new diagnostic challenge for clinical microbiology laboratories, as SARS-CoV-2 and influenza in particular have a significant impact on transmission inside and outside hospitals. Patients infected with SARS-CoV-2, Flu-A, Flu-B, HRSV, or other respiratory viruses have overlapping clinical presentations, but different approaches to treatment and patient management. Influenza viruses and HRSV have contributed to severe respiratory infections, that are a significant cause of morbidity, mortality and hospital admissions each year. Other respiratory viruses, such as human coronaviruses (HCoVs), human bocavirus (HBoV), human metapneumovirus (HMPV), parainfluenza viruses (PIV), human adenoviruses (HAdV), and enteroviruses (EV) are less important and less common than SARS-CoV-2, Flu-A or human rhinoviruses (HRVs). HRVs are also commonly detected in hospitalized patients of all ages and cause infections throughout the year with less seasonal variation. Without a definitive diagnosis, patients with viral respiratory infections are more likely to receive unnecessary antibiotic treatment. Therefore, rapid diagnosis combined with infection prevention measures is of paramount importance to stop the chain of transmission [4–7].

In such situations, multiplex rtRT-PCR, which enables the simultaneous detection of multiple respiratory viruses in a single tube with high throughput, provides a suitable solution. Several multiplex methods such as the FilmArray Respiratory Panel 2.1 (Bio-Fire Diagnostics, Salt Lake City, UT, USA), the XpertXpress SARS-CoV-2/Flu/HRSV assay (Cepheid, Sunnyvale, CA, USA) and the Respiratory Viruses (16 well) assay (AusDiagnostics, Mascot, Australia) are available for the simultaneous detection of a variety of respiratory viruses [4–11]. However, they are usually limited by throughput capacity, turn-around times (TAT) and/or cost. Consequently, these systems appear to be very well suited for use in small-scale testing, but not for high throughput testing. The Alinity m Resp-4-Plex assay (4-Plex) (Abbott, Chicago IL, US) represents a suitable method for both settings due to its high throughput capacity combined with rapid semi-random access and prioritisation of the samples and the ability to detect multiple targets in one reaction. Furthermore, the analytical and clinical performance of the 4-Plex assay has been extensively reviewed in the past and showed good overall performance [12–15].

The aim of this study was to assess the extent of undetected Flu-A/B and HRSV infections in early 2022, when the healthcare system was still focused on SARS-CoV-2 infections. Furthermore, the changes in the epidemiological patterns of Flu-A, FluB, and HRSV in the wake of the COVID-19 pandemic (winter 2022/2023) were analyzed. These results contribute to the knowledge of the impact of SARSCoV-2 on the epidemiology of Flu-A, FluB, and HRSV during and immediately after the pandemic and illustrate the usefulness of the tools acquired during the pandemic for the coming period.

## Methods

### Study time period selection

The study was conducted in two periods: period 1, from February to April 2022, an early 2022 period and period 2, from October 2022 to April 2023, a winter respiratory season 2022/23. Period 1 was chosen as the best time window to detect all viruses included in the 4Plex assay, if they were actually present in the population, due to the gradual lifting of COVID-19 prevention measures and, due to previous knowledge of the circulation of respiratory viruses in Slovenia. Period 2 was chosen based on to the fact that the COVID-19 preventive measures were no longer in place during the season of a respiratory infections in Slovenia [7]. This selection enabled us to compare the detected epidemiological pattern with the situation before the COVID-19 pandemic.

### Sample collection and testing protocol

In total, 20,049 patients were tested in the two selected time periods. In period 1, a total of 1,918 consecutively collected routine nasopharyngeal swabs were prospectively tested with the Alinity m SARS-CoV-2–specific assay (Abbott, Chicago IL, US) and in parallel also with the Alinity m 4Plex assay (Abbott, Chicago IL, US). There was no pre-selection of patients; all samples received during the selected study period were consecutively included in the study, with exception of already known SARS-CoV-2–positive patients who were sent for follow-up testing. In period 2, we prospectively tested 18,131 nasopharyngeal swab samples sent to the laboratory for routine respiratory infection diagnostic purposes, with the Alinity m 4-Plex assay.

### Preparation of the samples for the molecular testing

All samples were collected on the day of testing in a commercial 3 ml transport medium (VTM; Liofilchem, Roseto degli Abruzzi, Italy). The swabs were vortexed for 30 s at maximum speed (2,000 rpm) and 750 µl of the sample was used for the Alinity m 4-Plex Assay. In period 1, another 750 µl aliquot was tested on the same day without freezing and thawing using the Alinity m SARS-CoV-2 assay. The Alinity m system performed automated sample preparation, amplification, data acquisition and result interpretation.

### Data analysis and visualisation

Test results were exported from the MBL Laboratory Information System - LIS (Infonet, Slovenia) and analysed using Excel^®^ 2019 Version 1808 Build 10393.20026 (Microsoft, Redmont, IL) and R software Version 4.1.1 (The R Foundation, Indianapolis, IN). All results were linked to anonymised numbers, and only patient data on age, gender and type of healthcare facility where the sample was collected were used.

## Results

### Comparison of period 1 (gradual lifting of COVID-19 prevention measures) and period 2 (season 2022/23) according to age groups

The study group included 1,043/1,918 (54.4%) women and 875/1,918 (45.6%) men. Patients were further divided into five age groups; 0–3 years: 33/1,918 (1.7%) young children, 4–14 years: 121/1,918 (6.3%) children, 15–21 years: 113/1,918 (5.9%) young adults, 22–65 years: 1166/1,918 (60.8%) adults, 66 and over: 485/1,918 (25.3%) elderly.

In period 2, 9,084/18,131 (50.1%) women and 9,047/18,131 (49.9%) men were included. Patients were further divided into five age groups; 0–3 years: 766/18,131 (4.2%) young children, 4–14 years: 372/18,131 (2.1%) children, 15–21 years: 316/18,131 (1.7%) young adults, 22–65 years: 7,771/18,131 (42.9%) adults, 66 and over: 8,906/18,131 (49.1%) elderly.

The most affected group for Flu-A were young children in period 1, but in period 2 infections were more common in older children and young adults. Flu-B was detected in only two cases in period 1, but reappeared strongly in children in period 2. Human RSV was detected in all age groups in both periods. Most infections were present in young children in both periods, but incidence in the period 2 was much higher (3.0% vs. 26.4%). Also, in the elderly population the incidence of HRS was higher in period 2 (0.2% vs. 2.1%). SARS-CoV-2 infections dominated in all age groups in period 1 and remained present in all age groups in period 2, but the trend of infections moved towards the elderly.

**Table 1:** Comparison of the of positive results between the different age groups for period 1 and period 2

Age group	Young children (0 to < 4 y) No. (%)		Children (≥ 4 to < 15 y) No. (%)		Young adults (≥ 15 to < 22 y) No. (%)		Adults (≥ 22 to < 66 y) No. (%)		The elderly (≥ 65 y) No. (%)	
Virus	P1; n = 33	P2; n = 766	P1; n = 121	P2; n = 372	P1; n = 113	P2; n = 316	P1; n = 1,166	P2; n = 7,771	P1; n = 485	P2; n = 8,906
Flu-A	3 (9.1)	53 (6.9)	7 (5.8)	70 (18.8)	2 (1.8)	17 (5.4)	14 (1.2)	197 (2.5)	4 (0.8)	291 (3.3)
Flu-B	0 (0)	9 (1.2)	0 (0)	42 (11.3)	1 (0.9)	6 (1.9)	1 (0.1)	43 (0.6)	0 (0.0)	6 (0.1)
HRSV	1 (3.0)	202 (26.4)	2 (1.7)	11 (3.0)	1 (0.9)	3 (0.9)	9 (0.8)	101 (1.3)	1 (0.2)	188 (2.1)
SARS-CoV-2	21 (66.7)	40 (5.2)	63 (52.0)	25 (6.7)	64 (56.6)	28 (8.9)	743 (63.7)	606 (7.8)	376 (77.5)	1,039 (11.7)
Total	25 (75.8)	304 (39.7)	72 (59.5)	148 (39.8)	68 (60.2)	54 (17.1)	767 (65.8)	947 (12.2)	381 (78.6)	1,524 (17.1)

### Comparison of period 1 and period 2 according to the scale of testing and healthcare facilty

In period 1, the majority of samples, 1,531/1,918 (79.8%), originated from general population screening (outpatients) and 387 (20.2%) samples were from hospitals (inpatients) (Table 1). A total of 1,267 (66.1%) samples were SARS-CoV-2 positive, using the Alinity m SARSCoV-2 Single Plex Assay and the Alinity m 4-Plex Assay showing 100% agreement. Among the SARS-CoV-2 positive samples, co-detections were observed in 16/1,267 (1.3%) samples: SARSCoV-2 and Flu-A in 11/1,267 (0.9%) and SARSCoV-2 and HRSV co-detections in 5/1,267 (0.4%) samples. No other co-detections were observed. Of 651 respiratory samples that were negative for SARS-CoV-2, 19/651 (2.9%) samples were positive for Flu-A, 9/651 (1.4%) for HRSV, and 2/651 (0.3%) for Flu-B (Table 2).

**Table 2:** Detailed results of period 1 (early 2022), stratified by origin of samples (inpatient/outpatient) and SARS-CoV-2 status (positive/negative)

Category	Samples No. (%)	Flu-A positive No. (%)	Flu-B positive No. (%)	HRSV positive No. (%)
All samples	1,918 / 1,918 (100.0)	30 / 1,918 (1.6)	2 / 1,918 (0.1)	14 / 1,918 (0.7)
SARS-CoV-2 pos	1,267 / 1,918 (66.1)	11 / 1,267 (0.9)	0 / 1,267 (0.0)	5 / 1,267 (0.4)
SARS-CoV-2 neg	651 / 1,918 (33.9)	19 / 651 (2.9)	2 / 651 (0.3)	9 / 651 (1.4)
Inpatients total	387 / 1,918 (20.2)	5 / 387 (1.3)	1 / 387 (0.3)	5 / 387 (1.3)
SARS-CoV-2 pos	203 / 387 (52.5)	3 / 203 (1.5)	0 / 203 (0.0)	1 / 203 (0.5)
SARS-CoV-2 neg	184 / 387 (47.5)	2 / 184 (1.1)	1 / 184 (0.5)	4 / 184 (2.2)
Outpatients total	1,531 / 1,918 (79.8)	25 / 1,531 (1.6)	1 / 1,531 (0.07)	9 / 1,531 (0.6)
SARS-CoV-2 pos	1,064 / 1,531 (69.5)	9 / 1,064 (0.8)	0 / 1,064 (0.0)	4 / 1,064 (0.4)
SARS-CoV-2 neg	467 / 1,531 (30.5)	16 / 467 (3.4)	1 / 467 (0.2)	5 / 467 (1.1)

During period 2 (the 2022/23 season), a total of 18,131 samples were tested for respiratory viral infections. In total, 11,526/18,131 (63.6%) samples originated from inpatients and 6,605 (36.4%) from outpatients, as shown in detail in Table 3.

**Table 3:** Detailed results of period 2 (season 2022/23), stratified by the origin of sample (inpatient/outpatient) and SARS-CoV-2 status (positive/negative)

Category	Samples No. (%)	Flu-A positive No. (%)	Flu-B positive No. (%)	HRSV positive No. (%)
All samples	18,131/ 18,131 (100)	628 / 18,131 (3.5)	106 / 18,131 (0.6)	505 / 18,131 (2.8)
SARS-CoV-2 pos	1,738 / 18,131 (9.6)	15 / 1,738 (0.9)	1 / 1,738 (0.06)	13 / 1,738 (0.7)
SARS-CoV-2 neg	16,393 / 18,131 (90.4)	613 / 16,393 (3.3)	105 / 16,393 (0.6)	492 / 16,393 (0.3)
Inpatients total	11,526/ 18,131 (63.6)	195 / 11,526 (1.7)	26 / 11,526 (0.2)	186 / 11,526 (1.6)
SARS-CoV-2 pos	881 / 11,526 (7.6)	6 / 881 (0.7)	0/ 881 (0.0)	5 / 881 (0.6)
SARS-CoV-2 neg	10,645 / 11,526 (92.4)	189 / 10,645 (1.8)	26 / 10,645 (0.2)	181 / 10,645 (1.7)
Outpatients total	6,605 / 18,131 (36.4)	433 / 6,605 (6.6)	80/ 6,605 (1.2)	319 / 6,605 (4.8)
SARS-CoV-2 pos	857/ 6,605 (13.0)	9/ 857 (1.1)	1 / 857 (0.1)	8 / 857 (0.9)
SARS-CoV-2 neg	5,748 / 6,605 (87.0)	424 / 5,748 (7.4)	79 / 5,748 (1.4)	311 / 5,748 (5.4)

**Fig. 1:**
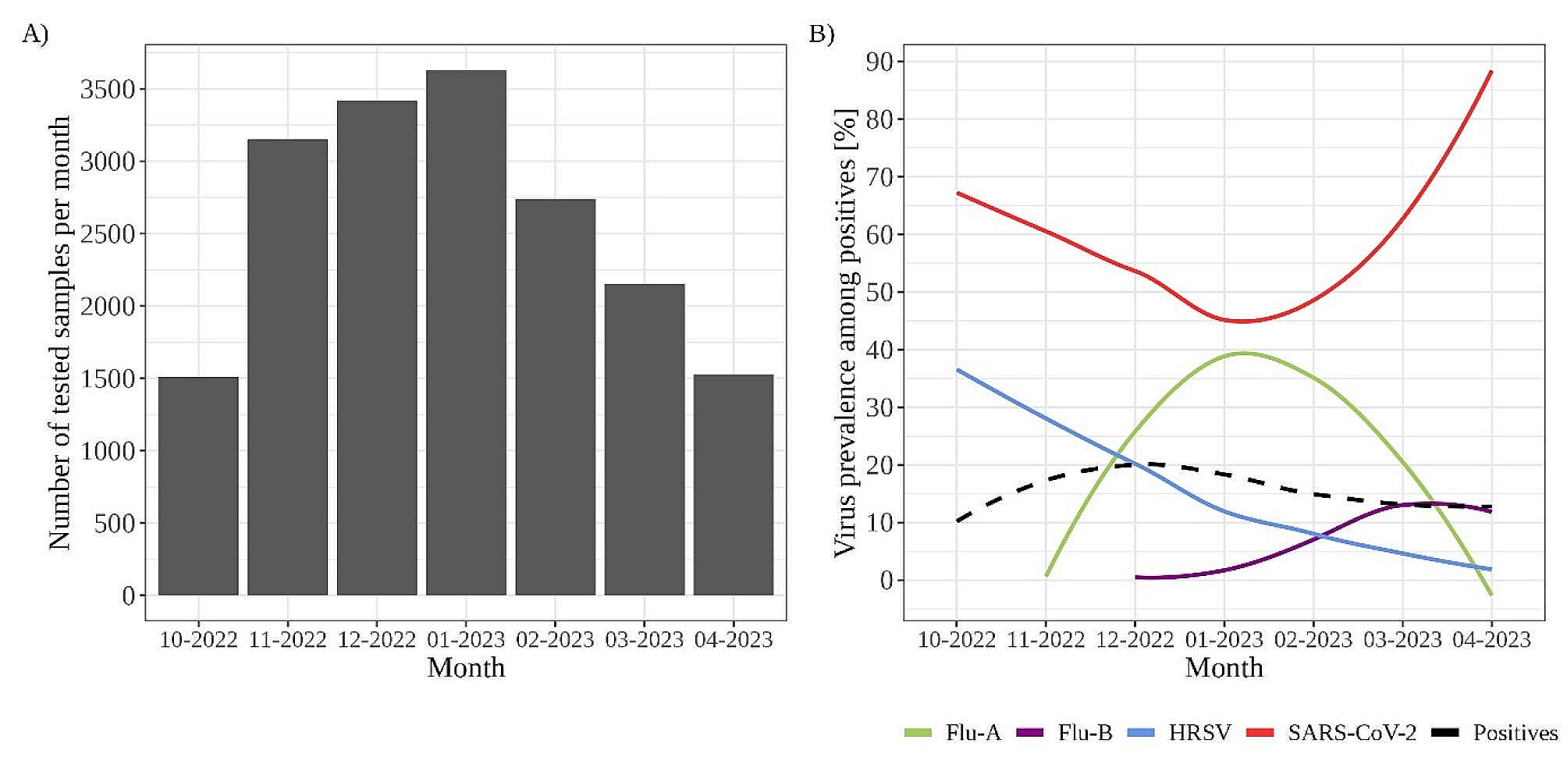
Number of samples tested per month in period 2 **(A)** and temporal occurrence **(B)** of SARS-CoV-2, Flu-A, Flu-B and HRSV in period 2, expressed as the proportion of each virus among all positive samples. The black dashed line represents the proportion of positive samples for all four viruses. The green, purple, blue and red lines represent the proportion of positive samples for each virus

A total of 3,521 (19.4%) samples were positive for one or more of the tested viruses (Flu-A, Flu-B, HRSV and SARS-CoV-2). The number of samples tested was not constant over the selected period. Most of the samples were tested between November 2022 and January 2023. In total, SARS-CoV-2 was detected in 1,738 (9.6%) samples, followed by Flu-A in 628 (3.5%), HRSV in 505 (2.8%), and Flu-B in 106 (0.6%) samples. SARSCoV-2 infections occurred throughout the respiratory season. The highest number of cases was observed in December 2022 (497 positive cases), followed by a gradual decline with a smaller peak in March 2023 (221 positive cases) (Fig. 1).

**Fig. 2:**
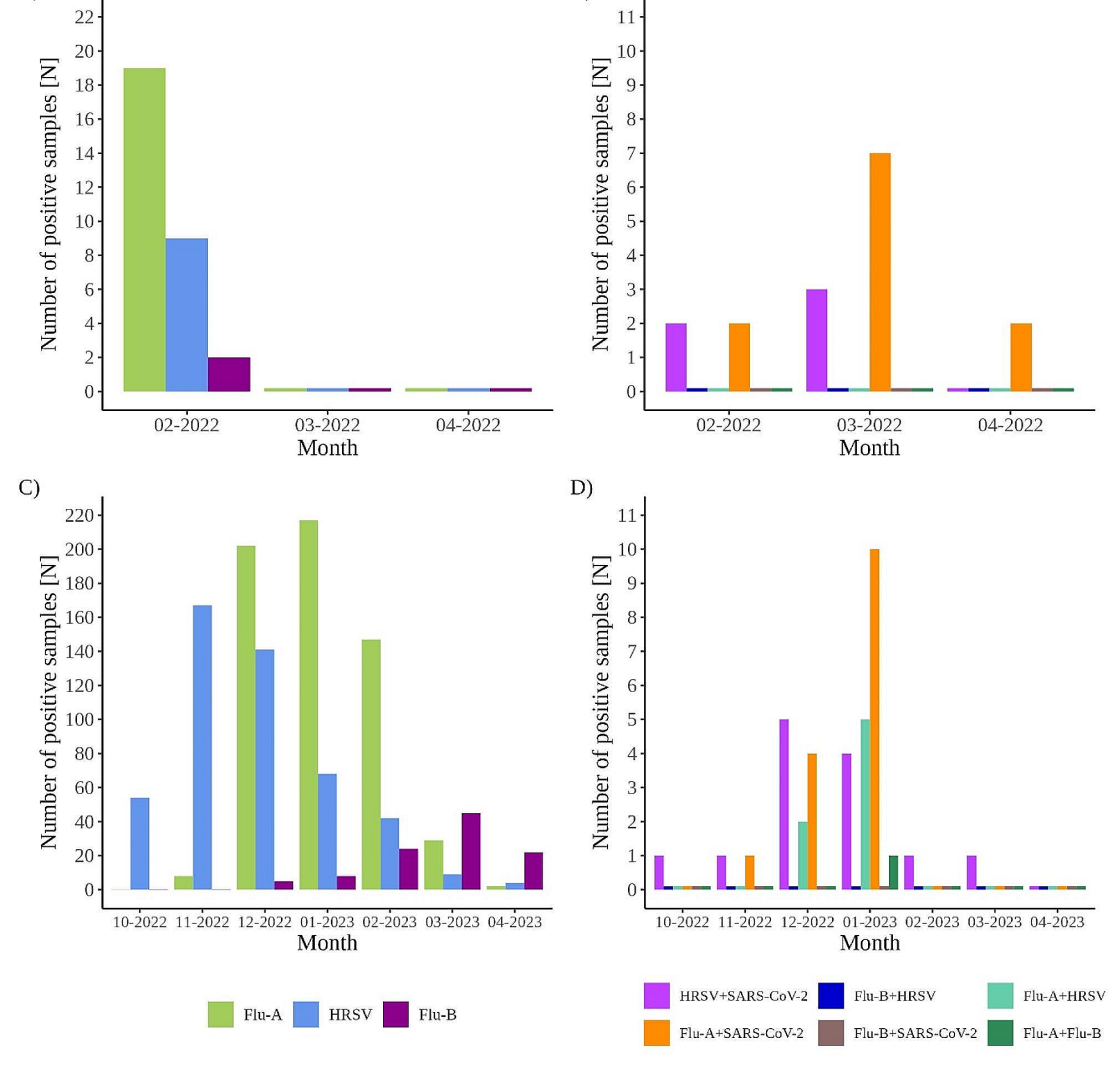
Temporal occurrence of positive samples from period 1 **(A, B)** and period 2 **(C, D).** Panels A and C represent the number of positive samples for Flu-A/B and HRSV. The number of positive samples for SARS-CoV-2 in each period is omitted from panels A and C, due to the high relative number compared to other viruses. The number of samples positive for SARS-CoV-2 is as follows (period 1: 02-2022 = 96, 03-2022 = 1043, 04-2022 = 112; period 2: 10-2022 = 105, 11-2022 = 297, 12-2022 = 497, 01-2023 = 297, 02-2023 = 127, 03-2023 = 221, 04-2023 = 165). Panels B and D represent the number of co-detections

Other viruses were detected more sequentially as successive single peaks with overlapping periods in between (Figs. 1 and 2). Human RSV was the first virus to peak in November 2022 (167 cases). It was gradually replaced by Flu-A, which peaked in January 2023 with 217 confirmed cases. The peak of Flu-B followed in March 2023, when 45 cases of Flu-B were detected (Figs. 1 and 2). The number of co-detections observed was similar for inpatients and outpatients. A total of 37 co-detections were observed in 2022/23 season. Most co-detections were between SARS-CoV-2/Flu-A in 15/18,131 (0.08%) samples, followed by SARS-CoV-2/HRSV in 13/18,131 (0.07%) samples, HRSV/Flu-A in 7/18,131 (0.04%) samples, and 1 sample we found SARSCoV-2/Flu-B and in another Flu-A/Flu-B co-detection (Fig. 2). A comparison of the overall results between the two periods is shown in Fig. 2.

**Fig. 3:**
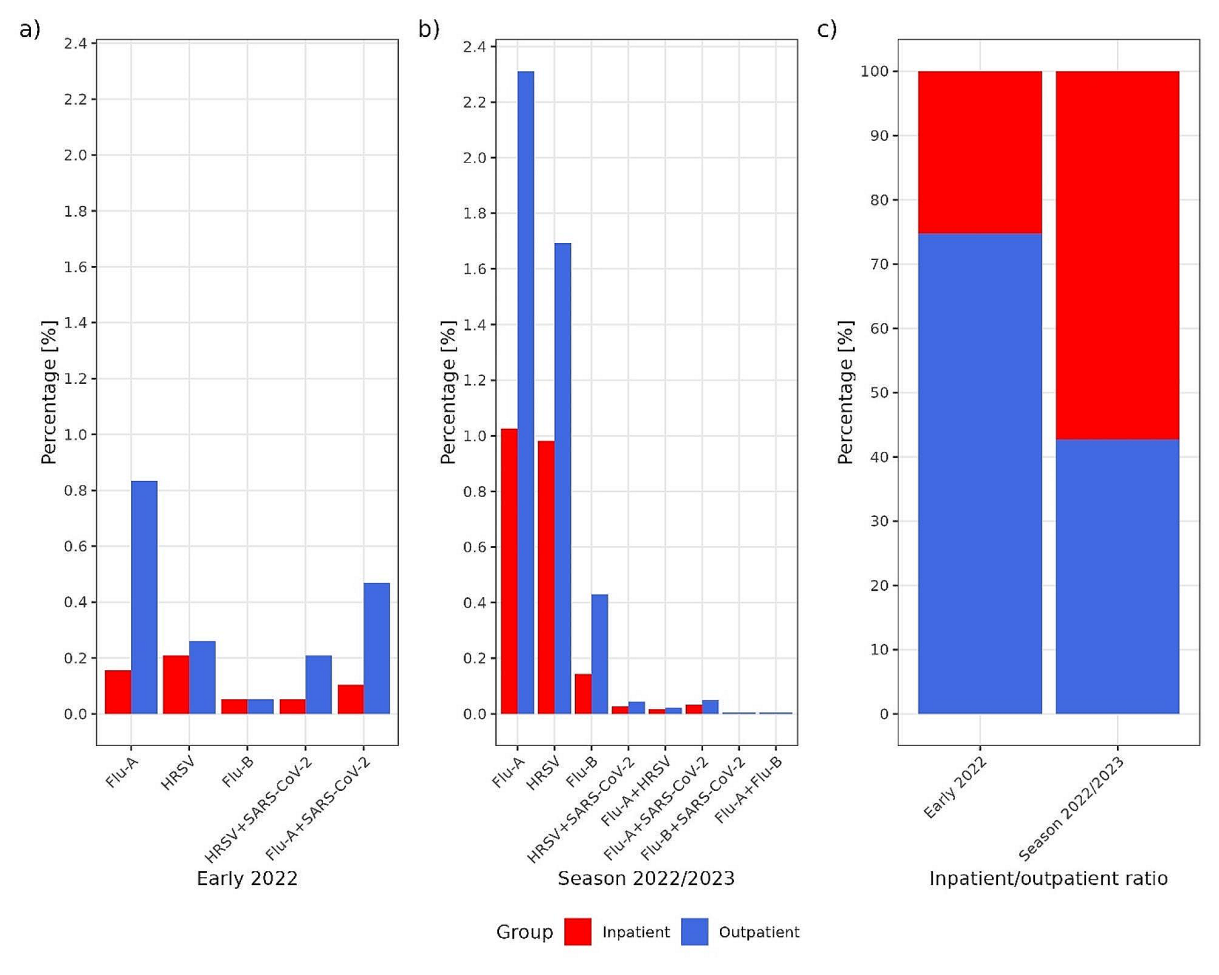
Comparison of positive inpatients (red) and outpatients (blue) to single and multiple infections except single SARS-CoV-2 infection, between period 1 **(a)** and period 2 **(b)**. Panel **(c)** shows a shift in the focus of testing from outpatients to inpatients between period 1 and period 2

## Discussion

In the study, we tested the performance of the 4-Plex multiplex respiratory panel assay on the Alinity m automated molecular system for the simultaneous detection of Flu-A, Flu-B, HRSV, and SARS-CoV-2 to determine the rate of overlooked Flu-A/B and HRSV infections in early 2022 and to investigate the epidemiological pattern of Flu-A/B and HRSV infections in the first “post-COVID-19” season. The Alinity m 4-plex was selected because it has good performance, high throughput, random access, semi-batch functionality, STAT (urgent) prioritization, and the ability to load and run multiple different assays simultaneously, making it suitable for both inpatient and outpatient diagnostics [11–15]. Full integration with the LIS enables automatic sample recognition, ordering of tests/markers per sample, and release of results, minimizing the possibility of errors.

We started the study already in February 2022 (period 1), when SARS-CoV-2 was still the most frequently detected virus (66.1%). Although the dominance of SARS-CoV-2 infections was undeniable, Flu-A (1.6%), HRSV (0.7%), and Flu-B (0.1%) were also detected, which would otherwise have been overlooked if the focus had been solely on the detection of SARS-CoV-2. These results suggest that the emergence of SARS-CoV-2 and the implementation of control measures has clearly impacted the prevalence of other respiratory viruses, as the detected infections with Flu-A, Flu-B, and HRSV were lower than in previous seasons, although the number of tests was higher [7]. In addition, the percentage of tested young children (1.7%) and the elderly (25.3%) in period 1, was lower than in period 2 (4.2% and 49.1%), which could also affect the lower number of influenza and HRSV cases in period 1. Prior to the emergence of SARSCoV-2, the epidemiology of respiratory viral infections in Slovenia followed an established pattern, with Flu-A/B, HCoVs, and HRSV infections peaking in the winter months and HAdV, HBoV, HMPV, and HRV detected throughout the year, with some EVs increasing during the summer [6, 16, 17]. The SARS-CoV-2 pandemic affected all respiratory viruses except HRVs, which were the second most frequently detected virus in hospitalised patients of all ages and the only viruses with unchanged seasonality [7]. Preventive measures were already in place and strictly followed between March 2020 and May 2021. No HRSV infection was detected during this time. After a one- year gap, HRSV cases reappeared in late spring and summer, with a peak in September 2021, suggesting that HRSV must have survived in the population, although it was clearly hampered by the pandemic and preventive measures [18]. Similarly, Flu-A and Flu-B infections in Slovenia were not detected during the 2020/21 season and only reappeared in March 2022 [7]. SARS-CoV-2 appears to be superior to other respiratory viruses in terms of resistance and infectivity [19]. Varela et al. (2021) reported that SARSCoV-2 was detected in 52.2% of samples, and only 0.4% of samples were positive for influenza in late spring and summer 2020. Human RSV also showed a significant decrease in the number of hospitalized cases with acute viral bronchiolitis compared to the previous respiratory season [20]. Infections with other respiratory viruses were also less common in the study by Uhteg et al. (2022), which was conducted in a period from October 2019 to December 2021 and ended just before the first period of this study. They found that the most common nonSARSCoV-2 infections were caused by FluA (10.1%), Flu-B (8.5%), and HRSV (5.4%). All available data suggest that COVID-19 prevention measures resulted in reduced transmission of influenza and HRSV, but were not sufficient to prevent the spread of the highly infectious SARS-CoV-2 virus [21].

In the second part of the present study (period 2), laboratory diagnostics of respiratory viruses in our institute slowly returned to a similar framework as before COVID-19. The origin of the samples also changed from screening of the general population back to targeted testing of inpatients (Fig. 3). It seems that the circulation of respiratory viruses has returned to the established pattern. Human RSV was the first virus to peak in November 2022. It was gradually replaced by Flu-A, which peaked in January 2023. The peak of Flu-B followed in March 2023. Compared to the previous part of the study, we detected a lower proportion of SARS-CoV-2 positive samples (9.6%) and an increase in cases of Flu-A (3.5%), HRSV (2.8%), and Flu-B (0.6%). We hypothesize that the number of SARS-CoV-2 infections has decreased due to the evolution of the virus (the Omicron variant replicates less efficiently than earlier variants and causes less cellular damage in infected cells [22]), global vaccination efforts, the substantial proportion of the population already exposed to the virus, and the lifting of COVID-19 control measures. The latter in particular, is leading to increased humantohuman contact, allowing other respiratory viruses to thrive again. A relative increase in influenza virus activity has already been reported in Australia compared to previous influenza seasons (2020/2021). Early surveillance data during the 2021/2022 influenza season in the Northern Hemisphere suggests that sporadic cases of influenza virus infections are re-emerging, but not at the same level of activity as before the COVID-19 pandemic. During the 2021/2022 influenza season, Flu-B virus predominated in China, while other Asian countries reported cases of both Flu-A and FluB [23]. During the 2022–2023 winter season, several countries in the Northern Hemisphere experienced an increase in influenza and HRSV infections. In Egypt, after two years of decline, a resurgence of influenza and HRSV was reported in children under 16 years of age. Higher infection rates were observed compared to pre-pandemic period [24].

Some observational data on the circulation of other respiratory viruses from routine diagnostics using the Respiratory Viruses (16 well) assay (AusDiagnostics) are available for both periods. In both periods, other respiratory viruses were intermittently detected, but their numbers were lower compared to SARS-CoV-2, Flu-A/B and HRSV, rarely exceeding 2.5%. The only exception was HRV, which was always present with little fluctuation. In period 2, HRV even became the predominant virus, with its highest prevalence recorded in October 2022 (25.2%). In the following months, HRV prevalence remained consistently high and never fell below 17.1%. (unpublished data). This suggests that with the Resp-4-plex assay we may miss infections with other viruses, although not in large numbers, except for HRV. However, the importance of such cases should not be underestimated and a two-step testing protocol could be considered as a solution to the problem. If the specimen is negative on the Resp-4-Plex assay, but the patient shows signs of a respiratory viral infection, the specimen should be considered for further testing with expanded respiratory virus detection test.Already in early 2022, when we detected SARS-CoV-2 in the majority of samples tested, codetections with other respiratory viruses were present in 1.2% of the samples analysed. Interestingly, a similar rate of co-detections was observed in the entire season 22/23 (1.7%). However, in period 2, co-detections other than SARS-CoV-2 were observed in 0.04% of the samples; HRSV/Flu-A co-detections occurred in 7 samples and Flu-A/Flu-B co-detection was observed in 1 sample. The estimates of co-detection in various cohort studies are also consistent with these results, ranging up to 3% [20, 25, 26]. On the other hand, some studies report higher rates of co-detection [25]. Kim et al. (2020) found that 20% of SARS-CoV-2 positive patients were also positive for another respiratory viral pathogen, with HRSV being the most common at 5.2% [27]. Swets et al. (2022) found co-detection with other respiratory viruses in 8.4% of patients, most commonly influenza and HRSV [28]. As indicated by the available literature, the extent of infections and co-detection with other respiratory viruses besides SARS- CoV-2 was low to moderate. To account for possible differences in virus detection according to patient age, both cohorts were divided into five age groups. The analysis showed a shift in the most affected group for Flu-A from young children in period 1 to children and young adults in period 2. Flu-B, which was detected in only two cases in period 1, reappeared strongly in children in period 2. HRSV was detected in all age groups in both periods, although it was strongly predominant in young children in period 2. Finally, SARS-CoV-2, which dominated in all age groups in period 1, remained present in all age groups in period 2, but with a trend towards the elderly. These results seem to indicate that when COVID-19 preventive measures were in place, other viruses lost their foothold in all age groups, but after the measures were relaxed, a similar epidemiological pattern is slowly being restored.

Few limitations of the study should be mentioned: since the included respiratory samples were selected sequentially as soon as they newly arrived at the laboratory and only SARS-CoV-2 follow-up tests were excluded, the selection of the study population might be biased. However, this effect is mitigated by a larger number of samples tested, which has not yet been done [3, 12–14]. Another limitation of this study is that we only tested for Flu-A/B, HRSV and SARS-CoV-2. It would be interesting to see the occurrence of other respiratory viruses, such as HRV and HAdV.

In conclusion, this study shows that infections with Flu-A/B and HRSV were relatively rare during the pandemic. The re-emergence of Flu-A/B and HRSV infections in the wake of the pandemic suggests that differential diagnosis will play a very important role in detecting and distinguishing SARS-CoV-2, Flu-A/B, or HRSV patients in the coming season. The ability to rapidly test for SARS-CoV-2, Flu-A, Flu-B, and HRSV on the Alinity m or similar analyzers will expand the ability to further increase testing capacity with the goal of containing the spread of viral respiratory infections other than just SARS-CoV-2.

## Data Availability

No datasets were generated or analysed during the current study.
